# Binocular Vision-Based Yarn Orientation Measurement of Biaxial Weft-Knitted Composites

**DOI:** 10.3390/polym14091742

**Published:** 2022-04-25

**Authors:** He Xiang, Yaming Jiang, Yiying Zhou, Benny Malengier, Lieva Van Langenhove

**Affiliations:** 1 Ministry of Education Key Laboratory of Advanced Textile Composite Materials, Institute of Composite Materials, Tiangong University, Tianjin 300387, China; he.xiang@ugent.be (H.X.); jiangyaming@tiangong.edu.cn (Y.J.); 2 School of Textile Science and Engineering, Tiangong University, Tianjin 300387, China; 3 Department of Materials, Textiles and Chemical Engineering, Ghent University, 9000 Gent, Belgium; benny.malengier@ugent.be (B.M.); lieva.vanlangenhove@ugent.be (L.V.L.); 4 Institute of Textiles and Clothing, The Hong Kong Polytechnic University, Hong Kong 999077, China

**Keywords:** binocular vision, textile composite, preform, yarn orientation, non-destructive testing, image processing

## Abstract

The mechanical properties of fiber-reinforced composites are highly dependent on the local fiber orientation. In this study, a low-cost yarn orientation reconstruction approach for the composite components’ surface was built, utilizing binocular structured light detection technology to accomplish the effective fiber orientation detection of composite surfaces. It enables the quick acquisition of samples of the revolving body shape without blind spots with an electric turntable. Four collecting operations may completely cover the sample surface, the trajectory recognition coverage rate reached 80%, and the manual verification of the yarn space deviation showed good agreement with the automated technique. The results demonstrated that the developed system based on the proposed method can achieve the automatic recognition of yarn paths of views with different angles, which mostly satisfied quality control criteria in actual manufacturing processes.

## 1. Introduction

In various industrial fields, fiber-reinforced polymers (FRPs) are more commonly used to develop load-bearing, lightweight products [1,2]. The key benefits are the ability to create complex shapes in a short manufacturing time while maintaining high specific mechanical properties [3]. In addition, it is critical to consider fiber orientation as a major factor in the entire technological process, because the FRP exhibits an anisotropic behavior that is mainly dependent on the fiber orientation.

All among the non-crimp fabrics, biaxial weft-knitted (BWK) fabrics, with excellent formability, flexible designability and low manufacturing costs, have been extensively used as reinforcements of composite materials in the automotive and aerospace industries [4,5,6]. However, there will be bending and shearing deformation of yarn during the fabric forming process, and the spacing between yarns will also change and lead to slippage, resulting in changes in fiber orientation and uneven distribution of the local fiber volume fraction [7,8]. The occurrence of the above phenomena will seriously affect the consistency of the final composite with the design goal [9,10]. Therefore, the detection of the fiber orientation after fabric forming is an essential factor to determine the mechanical properties of the composite components.

With the development of non-destructive testing (NDT) technology, researchers have carried out a lot of research work on the above problems by using different measurement methods [11,12]. El Said et al. [13] used computed tomography (CT) technology to analyze the local yarn orientation and corner bridging region after a preform forming procedure. However, the cost of CT technology is high, the imaging speed is slow, and it is difficult to detect structural parts with large or complex curvature. Wu et al. [14] characterized the fiber orientation and in-plane and out-of-plane waviness of carbon fiber composites based on eddy current testing technology. This method is applicable to large areas of composite structures and is able to deliver the local fiber orientation in the real state, but it can only be used to detect conductive materials—it is not applicable to insulating materials such as aramid fiber or glass fiber. Nelson et al. [15] showed how image processing methods can be used to create three-dimensional maps of ply orientations and waviness using ultrasonic instantaneous-phase data, but in practical processes, the sample must be soaked in water or sprayed with an ultrasound coupler on the sample surface before testing. Atkinson et al. [16] demonstrated the capabilities and limitations of polarization vision technology as applied to FRP component fiber angle inspections. During the image acquisition process, the sample cannot be moved and the sample shape is relatively flat. This method will result in blind spots when collecting samples with a rotary body or complex curvature, which lacks certain universality. Compared with the above methods, binocular vision detection technology can not only effectively obtain the depth information of the image, but also has advantages such as being extremely fast and cheap, and requiring very little physical space on an inspection/manufacturing line while maintaining competitive precision in comparison to the state of the art [17]. It has been widely used in defect detection, assembly positioning, size evaluation and other aspects in the field of composite material manufacturing [18]. However, we found no report on the application of this technology to yarn orientation detection after preform forming.

In the present study, a binocular vision system based on structured light for accurate yarn orientation detection is built. Integrating with an electric turntable, texture information and geometric shape information of the hemispherical shell structure BWK composite material are acquired without blind spots. The efficiency of the proposed method is analyzed systematically. The manually measured results of the yarn space are used to verify the accuracy of the method.

## 2. Materials and Methods

### 2.1. Experimental Sample

**Figure 1:**
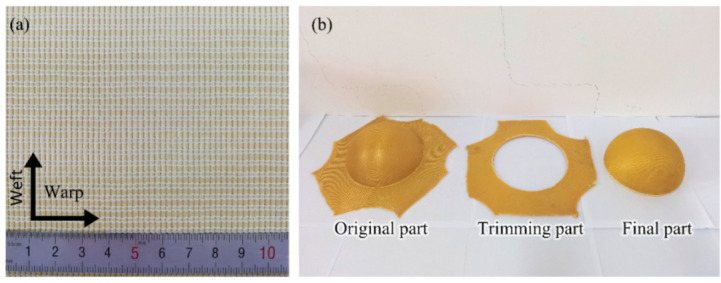
BWK fabric and composite specimens: (**a**) BWK fabric; (**b**) composite sample.

In this paper, aramid BWK fabric was used as the preform; both the warp and weft inserting yarns were made of Kevlar-49 aramid fiber tows, and the warp and weft densities were 4.7 tows/cm, as shown in Figure 1a. Only the weft inserting yarns and knitted loops can be seen from the top view, and the legs of the loop represent the direction of the warp inserting yarns [6]. The hemispherical shell was prepared by the vacuum infusion process (VIP). During the manufacturing process, one lay of fabric was formed on a female mold, which had a diameter of 150 mm. Then, vinyl ester resin R-806 was injected and cured under room temperature. After mechanical trimming, the final part was obtained, as shown in Figure 1b.

### 2.2. Experimental Setup

**Figure 2:**
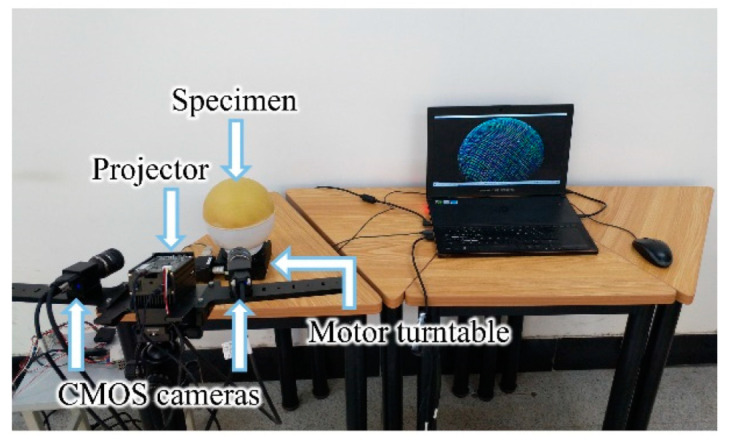
The binocular structured light 3D measurement system.

To realize the precise acquisition of 3D data, a full-view 3D data collection system (as shown in Figure 2) is built. The main equipment used for data acquisition includes two HIKVISION MV-CE013-80UM COMS cameras, two Computar MP1614-MP2 industrial camera lenses, a Tengju X20H structured light projector and a Sanying ERS100 electronic control turntable. The CMOS cameras are black-and-white industrial cameras with a resolution of 1280 × 1024 pixels. The focal length of the lenses is 16 mm. The resolution of the projector is 1280 × 720 pixels. The turntable’s diameter and resolution are 100 mm and 0.00125 deg, respectively.

The software platform was written in PCL, OpenCV within the C ++ environment, which realizes the functions of binocular system calibration, image processing and three-dimensional reconstruction of the yarn path. The system is around 500 mm away from the measured object when measuring the hemispherical specimen, and the angle between the two cameras is 60°. The measuring range of this system is approximately 320 × 250 mm, limited by the image resolution of the camera and the distance between the cameras and specimen.

### 2.3. Outline of Testing

**Figure 3:**
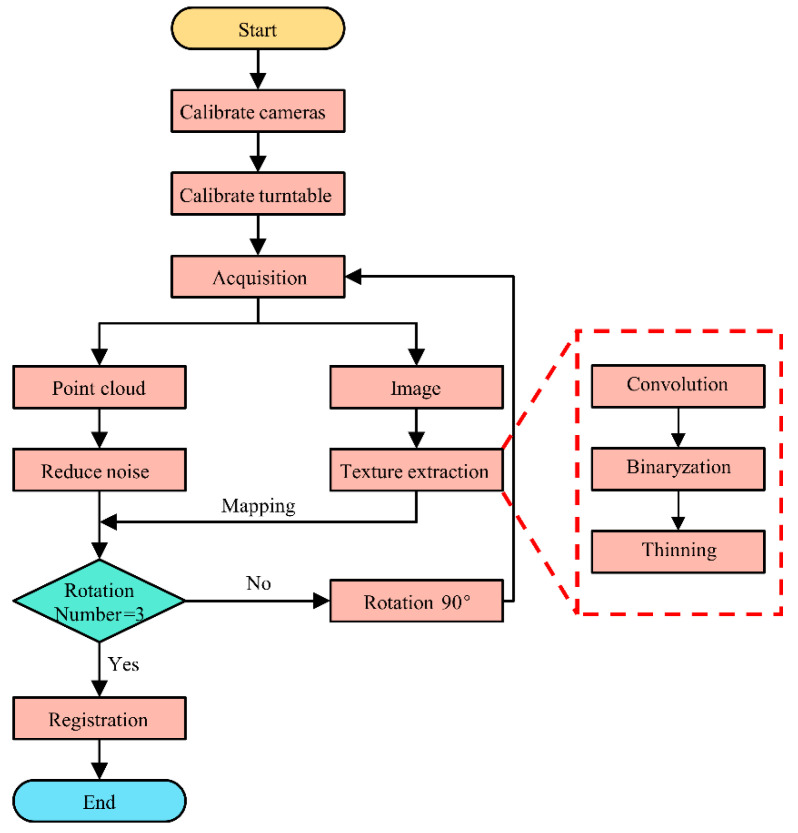
Flow chart of detection algorithm for yarn orientation.

The flowchart of the yarn orientation detection is shown in Figure 3. Firstly, we calibrate the binocular camera, and then obtain the location of the rotation axis of the turntable. Afterwards, the sample is placed on the spherical strut mold on the turntable for scanning, and the three-dimensional morphology information and image information of the sample part are acquired, respectively. After each acquisition step, the turntable rotates 90° and repeats the previous acquisition work. The image information collected each time will be filtered and its profile extracted. Next, this is mapped to three-dimensional space.

In the acquisition process, the rotating speed of the turntable is 5°/s, and the acquisition time of each camera is 6 s. The total operation time is 72 s.

## 3. Yarn Path Reconstruction

### 3.1. Stereo Calibration

**Figure 4:**
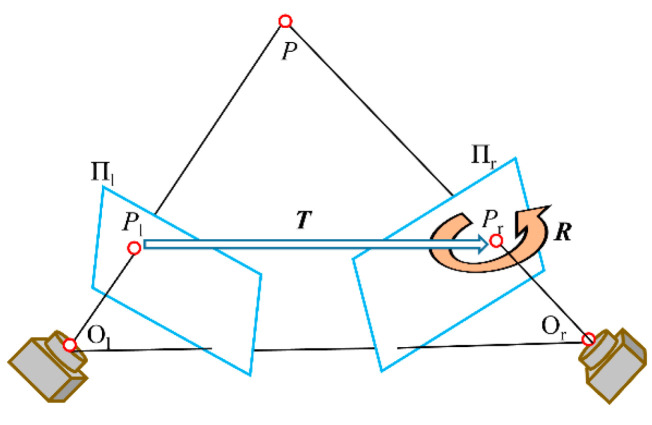
Principle of stereo calibration for binocular camera.

In this study, an improved Zhang’s calibration method proposed by Song et al. [19] is used to obtain the rotation matrix ***R***_l,_, ***R***_r_ and translation matrix ***T***_l_, ***T***_r_ from the world coordinate system (WCS) to camera coordinate system (CCS). These also include the internal and external parameters of the left and right cameras. In addition, it is also necessary to obtain the pose relationship of the two cameras relative to the same coordinate system through stereo calibration, i.e., rotation matrix ***R*** and translation matrix ***T***, so as to calculate the depth information of the point in the WCS [20]. The stereo calibration principle of the left and right cameras is shown in Figure 4.

After each acquisition process, the pixel point *P* in the WCS will be projected on the imaging planes Π_l_ and Π_r_ of the left and right cameras, respectively, and the points *P*_l_ and *P*_r_ are obtained; then,

(1) $$\{\begin{array}{c} P_{l}=R_{l}P+T_{l}\\ P_{r}=R_{r}P+T_{r} \end{array}\;$$

Taking the left camera as the reference, if the rotation and translation matrices between the left and right cameras are s are ***R*** and ***T***, the relationship between the matching points *P*_l_ and *P*_r_ is:

(2) $$P_{r}=RP_{l}+T\;$$

Combining Equations (1) and (2) gives:

(3) $$\{\begin{array}{c} R=R_{r}R_{l}^{-1}\\ T=T_{r}-RT_{l} \end{array}\;$$

### 3.2. Turntable Axis Calibration

In order to obtain the relationship before and after the rotation of a point around the axis, it is necessary to calculate the parameters of the turntable axis equation in the WCS and obtain the rotation angle. The calibration method adopted in this paper is as follows.

**Figure 5:**
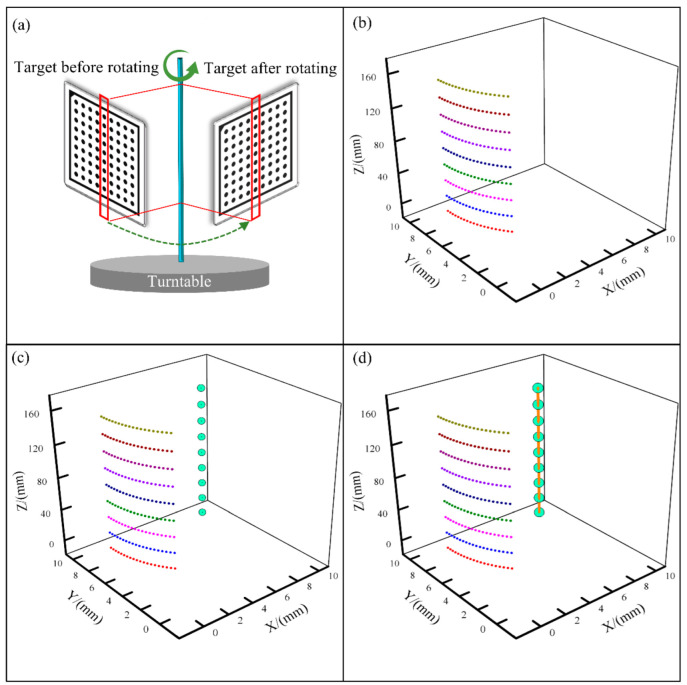
Calibration of turntable axis: (**a**) process of calibrating the turntable axis; (**b**) obtained point sets in different colors; (**c**) circle center fitting; (**d**) turntable axis fitting.

Firstly, a plane circular calibration target is vertically fixed on the turntable, and the rotating platform is controlled to drive the target to rotate. The target is acquired once every 2° of the turntable, and 20 times in total. Then, the motion trajectory of each tag on the target is theoretically a spatial circle centered on the axis of the turntable. The centers formed by the rotation of the tags at different positions should be located at different positions on the axis of the turntable. Finally, the position of the turntable axis in the WCS is obtained by fitting the positions of all circle centers. The solving process is shown in Figure 5. The specific implementation steps are as follows:

(1) According to the stereo calibration results, the point set of a column around the rotation axis in the CCS is acquired, as shown in Figure 5b;
(2) Calculate the centers of each motion trajectory formed by the rotation of a point around the turntable axis in point set ***P***, and the set of all the obtained centers is ***O***. These centers are located at different positions of the rotation axis, as shown in Figure 5c.
(3) The three-dimensional spatial line passing through the center point set ***O*** is fitted by the RANSAC method [21], which is the turntable axis, as shown in Figure 5d.

### 3.3. Acquisition of Three-Dimensional Data

**Figure 6:**
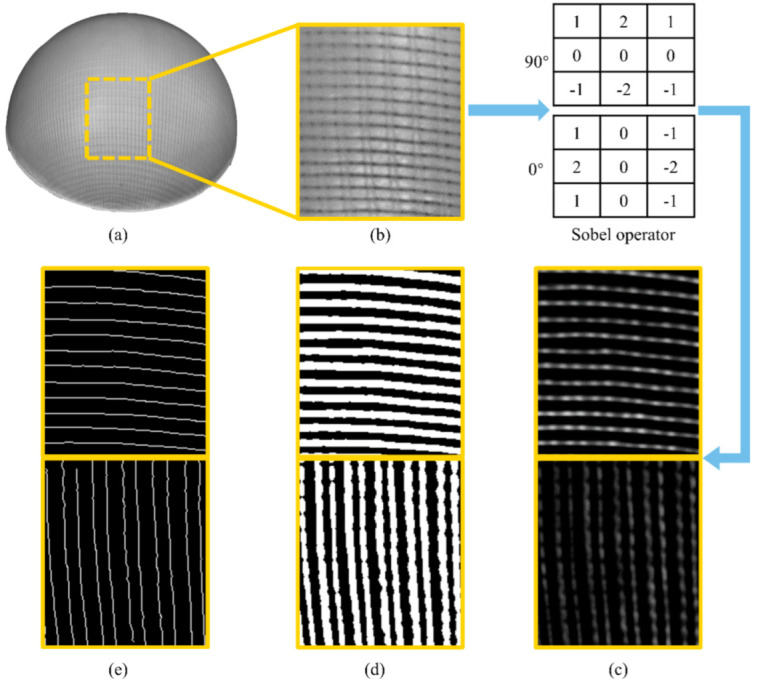
Image processing results: (**a**) original data; (**b**) close-up image of mean filter result; (**c**) convolution; (**d**) binarization; (**e**) thinning.

Firstly, the coded structured light is projected onto the object surface through the projector, and the image information of the object surface is acquired by the cameras. Then, the three wavelength phase shift profilometry method is used to decode the structured light to obtain the phase information [22]. Combined with the phase constraint and epipolar constraint, the three-dimensional point cloud data are generated. Because a black-and-white camera is used in this study, the point cloud data contain not only the spatial coordinate information of each pixel, but also the gray information with the value range of (0, 255), as shown in Figure 6a.

### 3.4. Feature Extraction

In order to extract the texture feature of yarn orientation, this study firstly uses the mean filtering method to remove the background and small useless features after obtaining the original image, as shown in Figure 6b. Conventionally, edge detection approaches use gradient differential operators such as the Roberts operator, Sobel operator, Prewitt operator and Canny operator [23]. Because of its reliability in analyzing noisy images, the 90° and 0° Sobel operators are utilized in the algorithms, so that the features of yarns in two directions are more prominent [24]. After this, the Gaussian filtering algorithm is used to enhance the visibility of the yarn contour (Figure 6c). Finally, the filtered contour is binarized, and the partially broken contour is connected by closing operation to obtain the complete path of the yarn, as shown in Figure 6d.

It can be found from Figure 6d that after binarization, each yarn contour contains too many pixels, resulting in a too wide yarn. Therefore, it is necessary to extract the skeleton of the yarn with a thinning algorithm to simplify the image data; the results are shown in Figure 6e.

### 3.5. Merging

**Figure 7:**
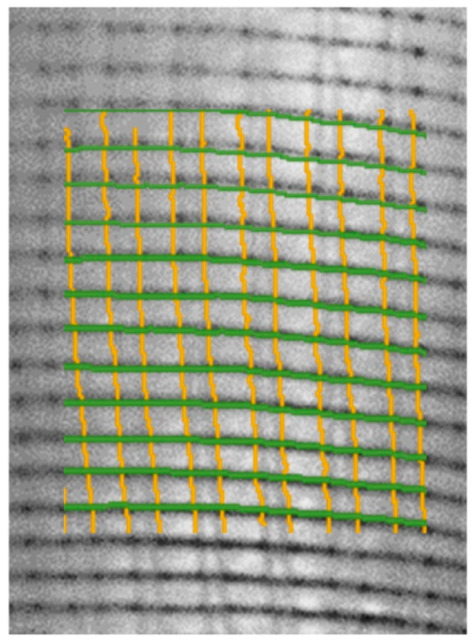
Close-up of the yarn edge detection results.

Since the thinned image contains binary data, the findcontour function in OpenCV can be directly used to extract the contour of the yarn. In this paper, contour data with a number of pixels of less than 30 are regarded as noise points and eliminated; moreover, the pixel information of each yarn is saved separately. In Figure 7, the results of the detected two directions are represented by green and yellow lines, respectively, and mapped on the original image.

Using the spatial coordinate information of each pixel from Section 3.3, the spatial path of each single yarn can be obtained, i.e., the two-dimensional data are mapped to three-dimensional data. At this time, the data acquired from the second to the fourth acquisition still need to be rotated to their correct location, by rotating around the axis of the turntable to merge in the correct position in the WCS [25]. It is assumed that the equation of the turntable axis obtained in Section 3.1 is Equation (4). The point P (*x*, *y*, *z*) and the rotation angle *θ* (*θ* = 90°, 180°, 270°) before rotating are known, and the coordinates of the rotated point P’ can be calculated by the matrix M, namely Equation (5).

(4) $$\frac{x-x_{0}}{a}=\frac{y-y_{0}}{b}=\frac{z-z_{0}}{c}$$

(5) $$M=\left[\begin{array}{cccc} a^{2}H+\cos\theta & abH-c\sin\theta & acH+b\sin\theta & \left(x_{0}-aK\right)H+\left(cy_{0}-bz_{0}\right)\sin\theta \\ abH+c\sin\theta & b^{2}H+\cos\theta & bcH-a\sin\theta & \left(y_{0}-bK\right)H+\left(az_{0}-cx_{0}\right)\sin\theta \\ acH-b\sin\theta & bcH+a\sin\theta & c^{2}H+\cos\theta & \left(z_{0}-cK\right)H+\left(bx_{0}-ay_{0}\right)\sin\theta \\ 0 & 0 & 0 & 1 \end{array}\right]$$

where H=1−cos(θ), $K=ax_{0}+by_{0}+cz_{0}$. The relationship between point P and P′ can be expressed by:

(6) $$\left[\begin{array}{c} x^{\prime }\\ y^{\prime }\\ z^{\prime }\\ 1 \end{array}\right]=M\left[\begin{array}{c} x\\ y\\ z\\ 1 \end{array}\right]$$

**Figure 8:**
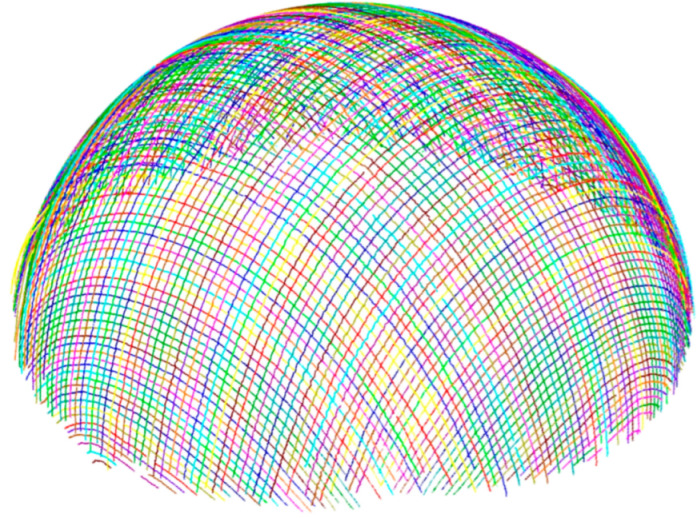
Overall reconstruction result.

The three-dimensional orientation reconstruction of the whole fabric can be realized by combining the spatial trajectory data of all yarns, as shown in Figure 8, where each yarn path is marked with a random color.

**Figure 9:**
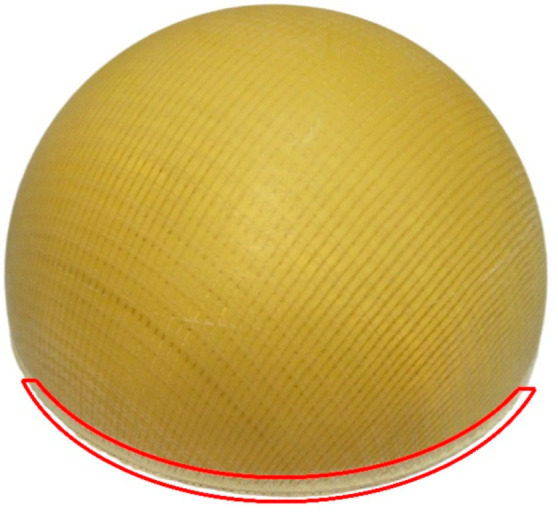
Local defects caused by mechanical cutting.

It can be seen from the reconstruction results that some of the yarns at the bottom edge of the shell have failed to reconstruct. This is due to the fact that when the hemispherical shell is mechanically cut, the resin at the processing place is subjected to force and microcracks form a white edge, which we have highlighted in the digital photo taken from the final part; see Figure 9. This white edge interferes with image acquisition and ultimately means that the yarn at the bottom edge of the shell is unable to be reconstructed.

## 4. Results and Verification

### 4.1. Trajectory Recognition Coverage Rate

In order to verify the feasibility of the system, the ratio of the yarn trajectory data coverage area to the pixel area of the original sample image is used as the ‘trajectory recognition coverage rate’ (TRCR) to evaluate the efficiency of yarn path extraction. In the 0° region, for instance, the detailed method works as follows.

**Figure 10:**
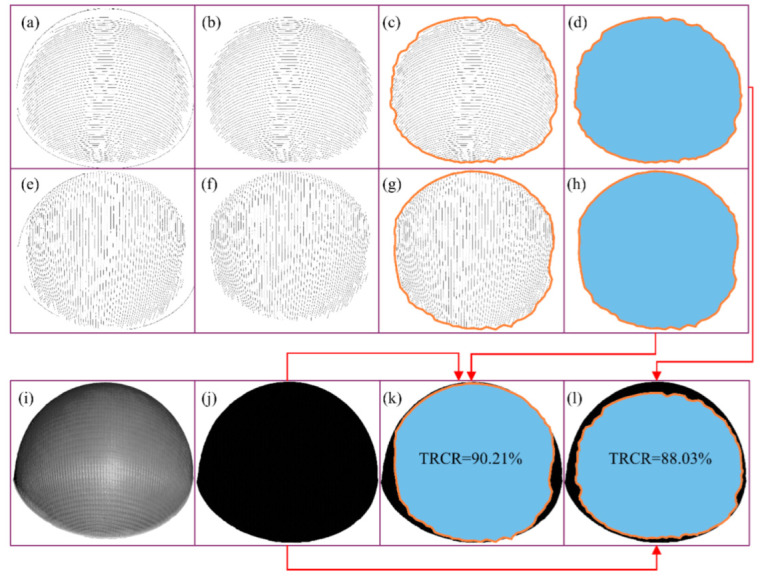
Calculation of the TRCR in reconstructed 0° region: (**a**) reconstruction result of weft yarn paths; (**b**) delete the edge contour of the sample from weft reconstruction result; (**c**) establish reconstruction area boundary in weft reconstruction result; (**d**) calculate the area of weft direction reconstruction area; (**e**) reconstruction result of warp yarn paths; (**f**) delete the edge contour of the sample from warp reconstruction result; (**g**) establish reconstruction area boundary in warp reconstruction result; (**h**) calculate the area of warp direction reconstruction area; (**i**) original image of sample; (**j**) binary image of sample; (**k**) TRCR result of warp yarn paths; (**l**) TRCR result of weft yarn paths.

Firstly, delete the pixels that represent the contour of the sample in the two-dimensional data by finding the most peripheral pixels (Figure 10b,f). Then, the outermost pixels are connected to form a closed region (Figure 10c,g), and the pixel area of yarn paths in the 0° region is calculated. The pixel areas of Figure 10d,h are 559,968 and 573,863 pixels, respectively.

Then, the sample’s image after removing the background is binarized, and the pixel area of the black pixel is calculated. For our example, this was 636,110 pixels, as shown in Figure 10j.

Finally, the TRCR values of the weft and warp direction are computed, which are 88.03% and 90.21%, respectively (Figure 10k,l). In the same way, the TRCR values of the 90°, 180°, 270° regions are shown in Table 1.

In addition, the area of the surface obtained by single scanning in Figure 10a can be obtained as 20,735.68 mm^2^ through the ‘Compute Area’ function of Geomagic software. One quarter of the hemisphere sample area is 8831.25 mm^2^; even with 86% TRCR, this system can completely reconstruct the yarn orientation of the sample after four times of acquisition.

### 4.2. Experimental Evaluation of Accuracy

The distance between yarns is an important factor for calculating the fiber volume friction of the composites. Thus, it was used to verify and evaluate the accuracy of the method by comparing the experimental results and scanned results.

**Figure 11:**
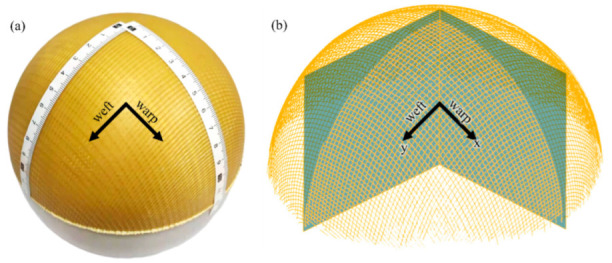
Accuracy evaluation of yarn space: (**a**) manually measuring yarn space; (**b**) obtaining the yarn space of the scanned data.

As shown in Figure 11a, the authentic yarn space was measured by sticking two paper rulers on the sample along the warp and weft direction from the top. For the scanned data, two feature planes along the warp and weft were built, as illustrated in Figure 11b. After this, the intersection points between the two planes and the paths of the warp and weft yarns were the objects for comparison.

**Figure 12:**
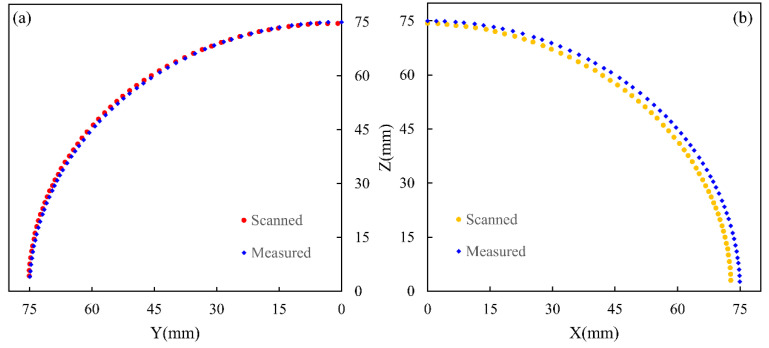
Yarn distance comparison results: (**a**) warp yarn profile location; (**b**) weft yarn profile location.

The results are shown in Figure 12, where the deviations of the yarn distance along the warp and weft directions are maximally 0.48 mm and 0.57 mm, respectively. It can also be seen that, since the manually measured data are the yarn space, its coordinates are located on the ideal sphere (with a radius of 75 mm). However, according to the scanned results, the radius of the sample along the warp direction becomes larger; on the contrary, it becomes smaller along the weft direction. This reflects sample yield distortion after the demolding process.

## 5. Conclusions

The aim of this paper is to provide a measurement method based on binocular vision for the characterization of yarn orientation in the BWK fabric-reinforced composite shell. The relevant conclusions can be stated as follows.

(1) A low-cost three-dimensional scanning system based on binocular structured light was built to realize the automatic, rapid and non-blind acquisition of three-dimensional data of the rotating sample. The three wavelength phase shift profilometry was used to reconstruct the three-dimensional morphology of the sample.
(2) The reconstruction results show that the TRCR reaches 86%. The assessment of the actual yarn space of the component shows a good correlation between the manual and scanning results. The measurement accuracy and coverage rate of the system have essentially met the quality control requirements of the practical production process.
(3) A drawback of this system is that in order to prevent the sample from moving during the rotation of the turntable, the rotation speed of the turntable used in this study is relatively slow. In the future, a firmer sample fixation method can be adopted and the rotation speed of the turntable can be increased, so as to further reduce the time-consuming nature of acquiring complete sample information.
(4) The main limitations with the approach outlined in this paper are that the sample shape should not have concavity so as to be fully visible to the camera. A solution to this could be to add another rotating axis of the sample holder. Moreover, this approach is limited to the analysis of the top (visible) layer of a part only.

Above all, the experimental results show that this method has remarkable value for equipment based on binocular vision technology to detect the yarn path in composite materials. At the same time, the parameters obtained by this method can be feasibly applied in the simulation practice of the composite forming process to improve the simulation accuracy and provide guidance for the actual manufacturing route.

## Figures and Tables

**Table 1:** The TRCR results of four regions.

Title 1	0°	90°	180°	270°
Weft	88.03%	86.71%	87.51%	87.78%
Warp	90.21%	91.47%	90.39%	91.49%

## Data Availability

Not applicable.
